# Exclusive or Partial Breastfeeding for 6 Months Is Associated With Reduced Milk Sensitization and Risk of Eczema in Early Childhood: The PATCH Birth Cohort Study

**DOI:** 10.1097/MD.0000000000003391

**Published:** 2016-04-18

**Authors:** Chih-Yung Chiu, Sui-Ling Liao, Kuan-Wen Su, Ming-Han Tsai, Man-Chin Hua, Shen-Hao Lai, Li-Chen Chen, Tsung-Chieh Yao, Kuo-Wei Yeh, Jing-Long Huang

**Affiliations:** From the Department of Pediatrics, Chang Gung Memorial Hospital at Keelung and Chang Gung University College of Medicine (C-YC, S-LL, K-WS, M-HT, M-CH); and Division of Pediatric Pulmonology (C-YC, S-HL) and Division of Allergy, Asthma, and Rheumatology, Department of Pediatrics (L-CC, T-CY, K-WY, J-LH), Chang Gung Memorial Hospital at Linkou, College of Medicine, Chang Gung University, Taoyuan, Taiwan.

## Abstract

There is insufficient evidence to confirm the association between breastfeeding and allergic outcomes later in life. This study aimed to determine the relationships between different breastfeeding patterns and allergen sensitizations and risk of developing atopic diseases in early childhood. A total of 186 children from a birth cohort in the Prediction of Allergies in Taiwanese Children study for a 4-year follow-up period were enrolled. Total serum immunoglobulin E (IgE) levels and specific IgE antibodies against food and inhalant allergens were measured sequentially at 6 months as well as at 1, 1.5, 2, 3, and 4 years of age. A significantly lower prevalence of milk sensitization was found in children at ages 1 and 1.5 years who were exclusively or partially breastfed for ≥6 months. Breastfeeding ≥6 months was significantly associated with a reduced risk of developing eczema but not allergic rhinitis and asthma at ages 1 and 2 years. Compared with exclusive breastfeeding ≥6 months, partial breastfeeding <6 months was significantly associated with an increased risk of developing eczema at ages 1 and 2 years. As with exclusive breastfeeding, partial breastfeeding for at least 6 months appears to be associated with a reduced prevalence of milk sensitization as well as a reduced risk of developing eczema in early childhood.

## INTRODUCTION

Breastfeeding provides nutritional and immunological protection against respiratory illnesses and gastrointestinal infections.^1^ However, the existing scientific evidence of the preventive effects of breastfeeding on atopic diseases in children is very limited.^2^ Exclusive breastfeeding for ≥4 months is reportedly associated with a decreased risk of asthma during childhood.^3,4^ However, infection-induced wheezing is common in early life and can easily be misdiagnosed as asthma. This protective effect of breastfeeding against asthma in early infancy may merely reflect a general protection against respiratory infections.^5^

One intervention trial reported that breastfeeding protected against atopic eczema.^6^ A longer duration of exclusive breastfeeding is reportedly associated with a lower risk for eczema in infants of mothers without allergy.^7^ However, a recent study reported an association between longer breastfeeding duration and an increased risk of eczema developing in high-risk infants.^8^ The relationship between breastfeeding and the development of atopic diseases remains controversial.

In infancy, allergic sensitization generally occurs to food allergens first. Early sensitization has consistently been identified as a risk factor for developing atopic diseases.^9,10^ Although exclusive breastfeeding for at least 4 months appeared to decrease the risk of milk sensitization in our previous study, no association was seen between exclusive breastfeeding and allergic outcomes later in childhood.^11^ Not all mothers are able to exclusively breastfeed their babies for the first 4 or 6 months for a variety of reasons. The effects of longer breastfeeding duration, even partial, on allergic sensitization and the development of atopic diseases have not been extensively studied.

The purpose of this study was to determine the impact of different breastfeeding patterns on the development of atopic diseases from birth to 4 years of age in children from a birth cohort in the Prediction of Allergies in Taiwanese Children (PATCH) study. The relationships between breastfeeding and sensitization to food and inhalant allergens were assessed and their relevance to the risk of developing atopic diseases later in childhood was examined.

## METHODS

### Study Population and Data Collection

The PATCH study included subjects from a birth cohort as well as those from cohorts of school and preschool children.^12^ Children who were recruited and completed 4 years of follow-up in a birth cohort study launched at Chang Gung Memorial Hospital (CGMH), Keelung from the year 2007 to 2010 were enrolled. Detailed descriptions of subject recruitment were reported previously.^10^ Details of information regarding demographic data, family atopy history, and the child's healthcare history including breastfeeding and medical conditions were collected. This study was approved by the Ethics Committee of CGMH (No. 102-1842C). All experiments in this study were performed in accordance with the relevant guidelines and written informed consent was obtained from the parents or guardians of all study subjects.

### Definition of Breastfeeding History

Breastfeeding can provide all of an infant's nutrition during the first 6 months of life as recommended by the World Health Organization.^13^ Detailed information on breastfeeding obtained by well-trained investigators about infants 6 months of age in combination with the breastfeeding status recorded routinely at outpatient clinics for immunization visits was used in the analysis. Specifically, mothers of the enrolled children were asked about their feeding habits. Infants who were fed breast milk only without additional foods or drinks except water were considered to be exclusively breastfed, while those who were fed formula only without additional foods or drinks except water were defined as formula fed. Those infants fed breast milk, formula, and other supplemental foods were considered partially breastfed.

### Evaluation and Diagnosis of Atopic Diseases

Information of allergic symptoms was obtained using the validated International Study of Asthma and Allergies in Childhood questionnaire.^14^ Atopic diseases were also evaluated by the same pediatric pulmonologist at outpatient clinics for a final diagnosis. The diagnosis of eczema was defined as a pruritic rash over the face and/or extremities with a chronic relapsing course.^15^ Allergic rhinitis was defined as a history of nasal allergy including sneezing, nasal congestion, itching, or rhinorrhea or the current use of medication for these symptoms.^16^ Asthma was diagnosed based on the Global Initiative for Asthma guidelines with the presence of a recurrent wheeze or current use of asthma medication.^17^ Infantile wheezing was defined as recurrent wheezing during the 1st year of life. Early-onset asthma was defined as asthma occurring before the age of 2.^18^

### Total and Allergen-Specific Serum Immunoglobulin E

Total serum immunoglobulin E (IgE) level was measured by ImmunoCAP (Phadia, Uppsala, Sweden), while allergen-specific IgE was determined using a commercial assay for IgE (ImmunoCAP Phadiatop Infant; Phadia). The most common food allergens (egg white, milk, and wheat) and inhalant allergens (*Dermatophagoides pteronyssinus*, *Dermatophagoides farina*, and *Cladosporium herbarum*) were selected and measured.^19^ Allergic sensitization was defined as ImmunoCAP Phadiatop Infant values ≥0.35 kU/L.^20^

### Covariates

Potential confounders associated with breastfeeding and atopic disease development were collected. Confounding variables related to atopic diseases such as child's sex, gestational age, maternal age at delivery, parental history of atopy, passive smoke exposure from parents during pregnancy, any older siblings, and household income were included and analyzed.

### Statistical Analysis

Differences were detected and comparisons of baseline characteristics among the different breastfeeding patterns were made using univariate parametric and nonparametric tests such as analysis of variance and the Kruskal–Wallis rank sum test, respectively. Standard binary logistic regression analysis methods were used to analyze the associations between breastfeeding and allergic sensitization and the risk of developing atopic diseases. Confounders related to the development of atopic diseases were included and adjusted in the multiple logistic regression analysis. The Statistical Package for the Social Sciences (SPSS Statistics for Windows Version 20.0; Armonk, NY) was used for the statistical analyses and GraphPad Prism Version 5.01 software (GraphPad Software Inc., San Diego, CA) was used to draw the graphs. All statistical hypothesis tests were 2-tailed and a *P* value < 0.05 was considered significant.

## RESULTS

### Population Characteristics

A total of 258 children were initially enrolled, but only 186 (72.1%) were regularly followed at the clinic for 4 years. The subject flow diagram of this birth cohort study was reported previously.^10^ Fear of blood draws and the parents’ unwillingness to attend regular outpatient follow-up visits were the major reasons for dropout.^10^ No significant difference was found in the baseline characteristics of these 186 children and the full 258 children, indicating that the 186 children could be considered a representative sample of the full cohort (see Table, Supplemental Content, which describes the comparison of basic characteristics of 186 children enrolled and the full 258 children in this cohort). The number of blood samples was collected differently at different ages; however, there was no significant difference in the prevalence of atopic diseases between children with and without blood samples during the follow-up period.^21^ Eczema, rhinitis, and asthma were diagnosed at the age of 4 years in 20, 58, and 26 children, respectively.

### Association Between Breastfeeding and Serum IgE Levels

**FIGURE 1:**
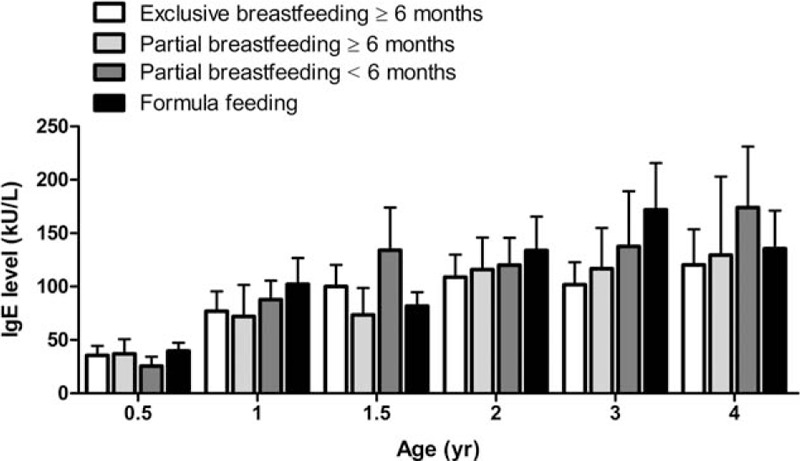
Changes of total serum IgE levels in children with different patterns of breastfeeding at different years of age. Data shown are mean ± standard error of the mean. IgE = immunoglobulin E.

Breastfeeding history was carefully reviewed in 186 children followed up at clinics for 4 years and the results were stratified into 4 groups: exclusive breastfeeding ≥6 months (n = 55, 29.6%); partial breastfeeding ≥6 months (n = 29, 15.6%); partial breastfeeding <6 months (n = 48, 25.8%); and formula feeding (n = 54, 29.0%). The children's characteristics by group are shown in Table 1. There was no difference in family history of allergic diseases among the different breastfeeding patterns. Children with the characteristics of paternal smoking had a higher incidence of receiving formula (*P* = 0.001). Figure 1 shows the changes in total serum IgE levels in children by group at different years of age. The total serum IgE levels fluctuated among different breastfeeding patterns but tended to increase with age until age 3. Children who were exclusively or partially breastfed for 6 months appeared to have lower serum IgE levels than those who were not. However, no significant difference was found between the different breastfeeding patterns and serum IgE levels across different ages.

**TABLE 1:**
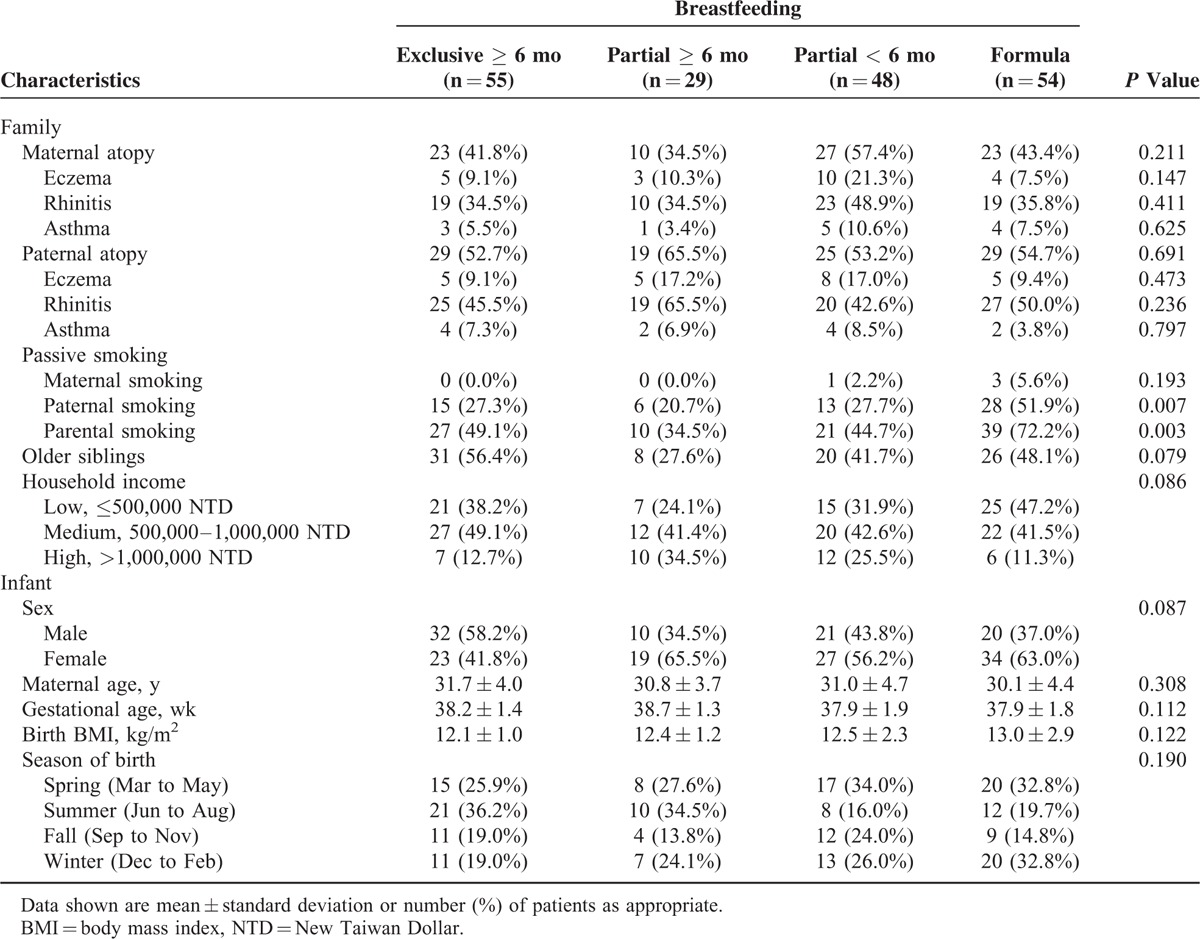
Baseline Characteristics of 186 Children in Relation to Different Patterns of Breastfeeding

### Association Between Breastfeeding and Allergen Sensitization

**FIGURE 2:**
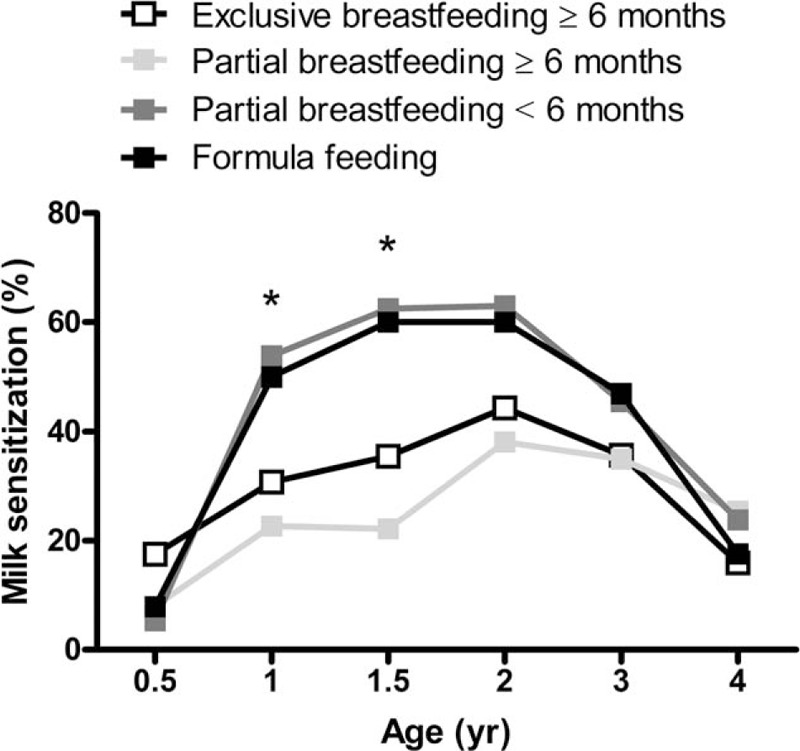
Prevalence of sensitization to milk categorized by different patterns of breastfeeding. Comparisons and differences of the prevalence of milk sensitization between children with different patterns of breastfeeding at different years of age. *P* values referred to the comparisons at ages 1 and 1.5 y are indicated by the marker. ^∗^*P* < 0.05.

An association analysis between breastfeeding and allergen sensitization showed that breastfeeding was significantly associated with milk sensitization but not egg white, wheat, or house dust mite sensitization. Comparisons and differences between the different patterns of breastfeeding and milk sensitization at different years of age are shown in Figure 2. A significantly lower prevalence of milk sensitization was found at ages 1 and 1.5 years in children who were exclusively or partially breastfed for ≥6 months compared with children who were formula fed or partially breastfed for <6 months.

### Association Among Breastfeeding, Milk Sensitization, and Atopic Diseases

Compared with formula feeding, exclusive breastfeeding (odds ratio, 0.37; 95% confidence interval [CI], 0.14–0.99; *P* = 0.049) and partial breastfeeding for ≥6 months (odds ratio, 0.19; 95% CI, 0.52–0.70; *P* = 0.012) were associated with a lower risk of milk sensitization at age 1.5 years. The association between breastfeeding, exclusive or partial, for ≥6 months and the risk of atopic diseases is shown in Table 2. Breastfeeding for ≥6 months was significantly associated with a lower risk of eczema but not allergic rhinitis or asthma at ages 1 and 2 years. Furthermore, compared with exclusive breastfeeding for ≥6 months, partial breastfeeding for <6 months was significantly associated with an increased risk of developing eczema at ages 1 and 2 years (Table 3).

**TABLE 2:**
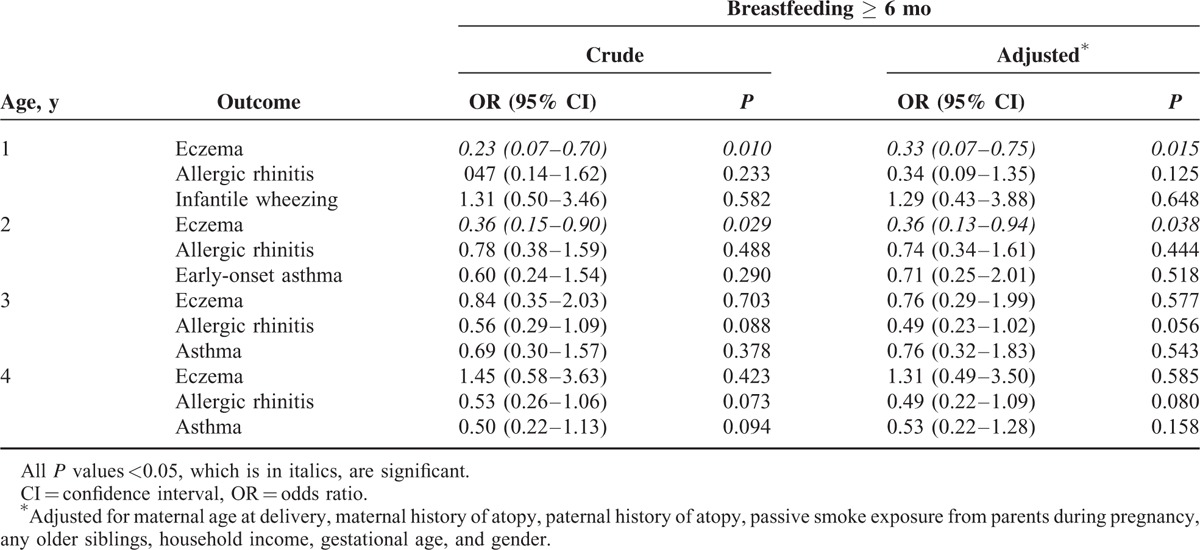
Association Between Breastfeeding, Exclusive or Partial, for 6 mo and the Risk of Atopic Diseases During Early Childhood

**TABLE 3:**
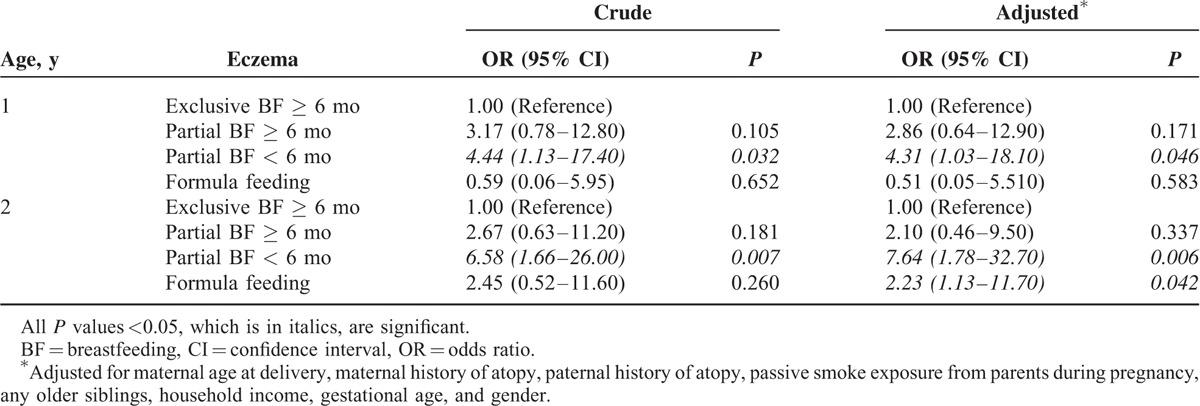
Association Between Different Patterns of Breastfeeding and the Risk of Eczema at Age 1 and 2

## DISCUSSION

Breastfeeding has been linked with improved infant and maternal health outcomes in the industrialized and developing world.^22^ Breastfeeding rates in Taiwan are on the rise, having increased from 5.4% in 1989 to 61.8% in 2010.^23^ However, in this study, the rates of exclusive and partial breastfeeding at 6 months postpartum were only 29.3% and 14.6%, respectively. Despite the widely recognized benefits of breastfeeding in Taiwan, the prolonged breastfeeding duration rate remains low. Furthermore, children who were breastfed longer breastfeeding were significantly more likely to not to be exposed to passive parental smoking in this study, indicating that mothers who breastfeed may also practice other health-promoting activities. This finding supports the need for health promotion activities to promote breastfeeding.^24^

IgE, a critical component of the adaptive immune system, plays a major role in allergic diseases.^19^ An allergic reaction occurs when the immune system overreacts to an allergen by producing IgE antibodies. The exclusive consumption of breast milk facilitates early maturation of the intestinal barrier and provides a passive barrier to potentially antigenic molecules.^22^ In this study, although there was no significant association between different breastfeeding patterns and serum IgE levels, children who were exclusively or partially breastfed for 6 months appeared to have relatively lower serum IgE levels. In addition, an increase in serum IgE levels is reportedly associated with increased sensitization to food allergens in early childhood.^10^ These findings suggest that breastfeeding may help prevent allergic reactions to food allergens and protect against allergies in early life.

Allergic sensitization is a known significant factor in the development of atopic diseases. Sensitization to food allergens is more common in early life and has consistently been identified as a risk factor for developing infantile eczema.^9,25^ In this study, breastfeeding for longer than 6 months appeared to be associated with both a significantly lower prevalence of sensitization to milk and a reduced risk of developing eczema until 2 years of age but not thereafter. The long-term effect of breastfeeding reportedly does not protect children against atopic diseases.^26,27^ The inverse association between longer breastfeeding and milk sensitization in early life observed in this study may explain the reports correlating breastfeeding and the reduced risk for developing atopic disease in early childhood only.

Several studies have reported that exclusive breastfeeding had a preventive effect on the development of atopic diseases early in life.^28,29^ The breastfeeding of high-risk infants to protect them against early wheezing illnesses is also recommended.^4^ However, recurrent wheezing in infants and young children is usually caused not only by allergies or asthma but also by frequent viral infections. In this study, there was no association between breastfeeding, sensitization to inhalant allergens, and the risk of developing asthma later in childhood. However, many reports have shown that prolonged and exclusive breastfeeding reduces the risk of infectious diseases in infancy.^1,30^ The findings in this study support the idea that the protective effect of breastfeeding against wheezing in early infancy may result from protection against respiratory infections.^5^

It is widely believed that prolonged breastfeeding and delaying the introduction of solid foods have benefits with regard to atopic dermatitis.^31^ Nevertheless, parental eczema is reportedly a major risk factor, and a longer breastfeeding duration may increase the risk of eczema in infants born to mothers with asthma.^8,32^ However, in this study, after the adjustment for a parental history of atopy, exclusive and partial breastfeeding for longer than 6 months were found to be still significantly related to a reduced prevalence of milk sensitization and provided protection against the development of eczema in early life. Our findings suggest the need for health-policy strategies to promote both exclusive breastfeeding and continued partial breastfeeding for preferably 6 months to not only reduce the prevalence of milk sensitization as well as reduce the development of eczema in early childhood.

There is strong evidence that breast milk provides multiple factors including humoral and cellular immunity components and that the biologically active molecules within it promote immune system maturation.^33^ Higher omega-3 essential polyunsaturated fatty acid and soluble CD14 concentrations in breast milk have been shown to correlate with a lower risk of eczema development.^34,35^ Breastfeeding also modulates the microbiota, and gut microbes may induce regulatory T cells involved in Th1/Th2 balance and enhance systemic innate immunity.^36,37^ These reports might explain the protective effects of breastfeeding against allergic diseases such as eczema observed in this study. However, future studies are needed to clarify the biological mechanisms underlying this association between breastfeeding and eczema.

One limitation of this study is its relatively small sample size with limited statistical power for the subanalyses. Furthermore, subclinical atopy may not have been diagnosed by physicians in this study's relatively short 4-year follow-up period. However, a significant strength of the present study lies in its longitudinal follow-up at very close intervals, which allowed for frequent sequential measurements of IgE levels, assessments of allergen sensitization, and accurate diagnosis of the presence of atopic diseases.

In conclusion, the rate of breastfeeding for at least 6 months remains low despite the widely recognized benefits of breastfeeding in Taiwan. Exclusive and partial breastfeeding for 6 months appears to decrease the prevalence of milk sensitization and protect against the development of eczema in early childhood. Although exclusive breastfeeding is recommended as the primary health strategy, continued partial breastfeeding for at least 6 months should also be encouraged to reduce milk sensitization and the risk of developing eczema in early childhood.

## Supplementary Material

Supplemental Digital Content
