# Supplementary material for: Exclusive or Partial Breastfeeding for 6 Months Is Associated With Reduced Milk Sensitization and Risk of Eczema in Early Childhood: The PATCH Birth Cohort Study
Source: Medicine (Baltimore). 2016 Apr 18;95(15):e3391. doi: 10.1097/MD.0000000000003391 (PMC4839855; doi:10.1097/MD.0000000000003391)
Supplement: Supplemental Digital Content [file medi-95-e3391-s001.doc]

**Supplemental Table.** Comparison of basic characteristics of 186 children enrolled and the full 258 children in this cohort.

| Characteristics | Enrolled  (n = 186) | Total  (n = 258) | *P*-value |
| --- | --- | --- | --- |
| **Family** |  |  |  |
| Maternal atopy | 77 (43.0%) | 97 (37.7%) | 0.269 |
| Eczema | 22 (12.3%) | 22 (8.6%) | 0.203 |
| Rhinitis | 66 (36.9%) | 85 (33.1%) | 0.412 |
| Asthma | 12 (6.7%) | 14 (5.4%) | 0.586 |
| Paternal atopy | 94 (52.5%) | 113 (44.0%) | 0.079 |
| Eczema | 21 (11.7%) | 14 (8.6%) | 0.348 |
| Rhinitis | 83 (46.4%) | 92 (38.3%) | 0.099 |
| Asthma | 10 (5.6%) | 11 (7.1%) | 0.571 |
| Passive smoking |  |  |  |
| Maternal smoking | 4 (2.2%) | 13 (5.1%) | 0.134 |
| Paternal smoking | 62 (34.6%) | 93 (36.2%) | 0.739 |
| Parental smoking | 64 (35.8%) | 98 (38.1%) | 0.613 |
| Older siblings | 81 (45.3%) | 117 (45.5%) | 0.955 |
| Household income |  |  | 0.938 |
| Low, ≤ 500,000 NTD | 65 (36.3%) | 95 (37.0) |  |
| Medium, 500,000-1,000,000 NTD | 83 (46.4%) | 115 (44.7%) |  |
| High, > 1,000,000 NTD | 31 (17.3%) | 47 (18.3%) |  |
| **Infant** |  |  |  |
| Sex, male | 80 (44.4%) | 131 (50.8%) | 0.192 |
| Maternal age (yr) | 30.6 ± 4.4 | 30.5 ± 4.6 | 0.851 |
| Gestational age (wk) | 38.1 ± 1.7 | 38.1 ± 1.8 | 0.980 |
| Birth BMI (kg/m^2^) | 12.5 ± 2.2 | 12.5 ± 2.0 | 0.720 |
| Season of birth |  |  | 0.534 |
| Spring (Mar to May) | 59 (32.8%) | 74 (28.8%) |  |
| Summer (Jun to Aug) | 47 (26.1%) | 59 (23.0%) |  |
| Fall (Sep to Nov) | 28 (15.6%) | 48 (18.7%) |  |
| Winter (Dec to Feb) | 46 (25.6%) | 76 (29.6%) |  |

Data shown are mean ± SD or number (%) of patients as appropriate. NTD, New Taiwan Dollar; yr, year; wk, week; BMI, body mass index.
